# Genetic compensation in a stable *slc25a46* mutant zebrafish: A case for using F0 CRISPR mutagenesis to study phenotypes caused by inherited disease

**DOI:** 10.1371/journal.pone.0230566

**Published:** 2020-03-24

**Authors:** Elena Buglo, Evan Sarmiento, Nicole Belliard Martuscelli, David W. Sant, Matt C. Danzi, Alexander J. Abrams, Julia E. Dallman, Stephan Züchner

**Affiliations:** 1 John P. Hussman Institute for Human Genomics, University of Miami Miller School of Medicine, Miami, Florida, United States of America; 2 Department of Biological Sciences, University of Miami, Coral Gables, Florida, United States of America; 3 Department of Biomedical Informatics, University of Utah, Salt Lake City, Utah, United States of America; 4 Department of Genetics, Yale University, New Haven, Connecticut, United States of America; Medical College of Wisconsin, UNITED STATES

## Abstract

A phenomenon of genetic compensation is commonly observed when an organism with a disease-bearing mutation shows incomplete penetrance of the disease phenotype. Such incomplete phenotypic penetrance, or genetic compensation, is more commonly found in stable knockout models, rather than transient knockdown models. As such, these incidents present a challenge for the disease modeling field, although a deeper understanding of genetic compensation may also hold the key for novel therapeutic interventions. In our study we created a knockout model of *slc25a46* gene, which is a recently discovered important player in mitochondrial dynamics, and deleterious mutations in which are known to cause peripheral neuropathy, optic atrophy and cerebellar ataxia. We report a case of genetic compensation in a stable *slc25a46* homozygous zebrafish mutant (hereafter referred as “mutant”), in contrast to a penetrant disease phenotype in the first generation (F0) *slc25a46* mosaic mutant (hereafter referred as “crispant”), generated with CRISPR/Cas-9 technology. We show that the crispant phenotype is specific and rescuable. By performing mRNA sequencing, we define significant changes in *slc25a46* mutant’s gene expression profile, which are largely absent in crispants. We find that among the most significantly altered mRNAs, *anxa6* gene stands out as a functionally relevant player in mitochondrial dynamics. We also find that our genetic compensation case does not arise from mechanisms driven by mutant mRNA decay. Our study contributes to the growing evidence of the genetic compensation phenomenon and presents novel insights about Slc25a46 function. Furthermore, our study provides the evidence for the efficiency of F0 CRISPR screens for disease candidate genes, which may be used to advance the field of functional genetics.

## Introduction

Genetic compensation (genetic buffering) is a phenomenon in which an organism with a pathogenic mutation does not develop the expected adverse phenotype due to compensatory actions of another gene or genes, which functionally compensate for the loss-of-function genotypes, restoring more normal physiological function. The first evidence of genetic compensation was reported in 1932 in the form of dosage compensation in Drosophila. Male flies showed an up-regulated transcription from their X chromosome, resulting in similar gene expression as in female fruit flies, which possess two X chromosomes [1]. Since then genetic compensation has been shown to be a wide-spread phenomenon having been reported in diverse phyla including fly[1–5], plants [6, 7], yeast[8], zebrafish[9–12], and mice[13–15]. There is also evidence from genome-wide association studies suggesting that genetic compensation, by way of genetic modifiers, may explain the absence of affected status in some humans carrying deleterious mutations [16, 17], and thus is of great interest for potential medical applications.

Genetic compensation may result from diverse mechanisms, including: upregulation of genes with redundant functions, or from a more complex response within metabolic, signaling or transcriptional networks. Compensation may be result from an upregulation of a paralog gene[10], an action of a genetic modifier, or an orchestra of genetic changes[18], which may contribute to the same biological process[19]. Overall, the mechanisms of genetic compensation are not fully understood. There is also a range of terminological definitions, which, for the clarity of our conceptual interpretations, we define for this manuscript in Table 1.

**Table 1:** Term definitions.

Genetic compensation (genetic buffering)	A broad phenomenon that encompasses diverse changes in gene expression and/or variability in genetic background, which phenotypically compensate for loss-of-function genotypes by versatile mechanisms: via upregulation of gene paralogs, via action of genetic modifiers, or complex responses within functional gene networks
Transcriptional adaptation	Changes in RNA levels that result from a genetic mutation and not from the loss of gene function, mechanistically driven by nonsense-mediated decay[19]
Genetic modifier	A genetic allele, which does not possess sequence homology with the loss-of-function gene, and is able to provide phenotypic compensation within a loss-of-function genotype by serving compensatory roles towards the same biological process, not necessarily as a response to a deleterious mutation

Recently, reports of discrepancies between knockdown and mutant phenotypes have increased, many of which were observed in zebrafish[20–25]. Zebrafish are a versatile convenient vertebrate model system with many advantages for reverse genetics, such as simplicity of genetic manipulation, fast generation times of large numbers of progeny, and optical transparency suitable for live imaging[26]. Thus, absence of stable mutant phenotypes and inconsistencies with the knockdown morpholino-induced phenotypes have raised concerns in the functional genetics field that is aimed at understanding disease symptom etiology. These discrepancies have been attributed to nucleotide toxicity and off-target effects of the morpholino [22, 25]. Conversely, recently published experiments in several zebrafish mutant models demonstrate that at least in some cases the absence of the phenotype is due to genetic compensation[10–12]. One recently discovered mechanism involves the upregulation of either gene paralogs or genes with sequence homology, triggered by nonsense-mediated-decay (NMD) of the mutant mRNA, also referred as transcriptional adaptation response[9, 19]. In the long-run, a more thorough understanding of genetic buffering may uncover therapeutic strategies, as well as inform inherited disease modeling strategies.

Here we address disease modeling approaches through the lens of the disease gene *SLC25A46*. Human recessive loss of *SLC25A46* function causes a spectrum of disorders that range from optic atrophy to Charcot-Marie-Tooth type 2, Leigh syndrome, progressive myoclonic ataxia and lethal congenital pontocerebellar hypoplasia[27–32]. Two mouse *Slc25a46* mutant models have shown to develop cerebellar ataxia, optic atrophy, peripheral neuropathy[33], and neuromuscular junction defects[34]. Another group reported *Slc25a46* knockout mouse with altered mitochondrial network in the peripheral nerves, hypoglycemia, ataxic gait, muscle loss, smaller internal organs and shorter life span[35]. The zebrafish knockdown model was assessed in early larval developmental stages and showed optic nerve maldevelopment and disrupted primary motor neuron axons at 48 hours post fertilization (hpf)[30, 31]. Despite these models, it remains unclear how loss SLC25A46 function causes symptoms of disease.

SLC25A46 is an atypical member of mitochondrial carrier SLC25 family. This family of proteins is involved in transporting keto acids, amino acids, nucleotides, inorganic ions and co-factors across the mitochondrial inner membrane[36]. Its members share the same structural folds, consisting of six trans-membrane alpha-helices and three matrix helices, arranged with threefold pseudo-symmetry[36, 37]. Although SLC25A46 is a member of the solute carrier family, its exact transport function is still not known and is possibly rudimentary. Unlike most members of the SLC25 family, SLC25A46 localizes to the outer mitochondrial membrane and was shown to function as a player in mitochondrial dynamics: a pro-fission protein interacting with Mitofusin 2 (*mfn2*), optic atrophy-1 (*opa1*), and mitochondrial cristae organizing system (MICOS) complex[38, 39]. SLC25A46 has also been shown to be important in maintaining mitochondrial architecture and the shape of cristae[38]. In cell culture, *SLC25A46* knockdown has been shown to result it hyperfused mitochondria, while its overexpression leads to fragmented mitochondria[31]. Additionally, SLC25A46 was also shown to function in lipid transfer between endoplasmic reticulum (ER) and mitochondria[38]. Therefore, SLC25A46 has diverse functions that are incompletely understood.

In the present study we used CRISPR/Cas9 gene-editing technology in zebrafish to generate both an F0 mosaic model and an F0-derived stable homozygous *slc25a46* mutant model, so that we could better understand Slc25a46 function and disease mechanisms[40]. In contrast to morpholino, CRISPR induces permanent changes in organism’s DNA, and due to its stochastic gene editing in dividing cells of the developing organism it results in a mosaic genotype when animals are first mutagenized[40–43]. Such an F0 mutant has the potential to approximate the stable mutant genotype, yet with significant advantages over the knockdown morpholino models that include minimal cytotoxicity and the opportunity to assess mutant phenotypes throughout life span in the first generation. As such, generation of F0 mutants is an attractive approach to explore symptom etiology in disease models that bypasses the burden of months and years of animal husbandry and time required for genotyping and confirming a stable mutant line. By comparing F0 crispants and multigenerational *slc25a46* mutant larvae, we find phenotypic compensation in the stable *slc25a46* mutant. In contrast, mosaic F0 crispants do not exhibit genetic compensation or replicate the robust and rescuable phenotypes which were previously reported in the *slc25a46* morpholino knockdowns. RNA sequencing shows that the absence of a phenotype in *slc25a46* mutants is associated with significant changes in expression of candidate compensatory genes. Our study adds to the growing body of evidence of diverse mechanisms of genetic buffering phenomena. Importantly, we present evidence in support of the F0 CRISPR targeting approach, which gives promise of fast and efficient confirmation of disease genes in a vertebrate animal model of inherited disease.

## Results

### CRISPR targeting of *slc25a46* induces a severe genetic lesion

**Fig 1:**
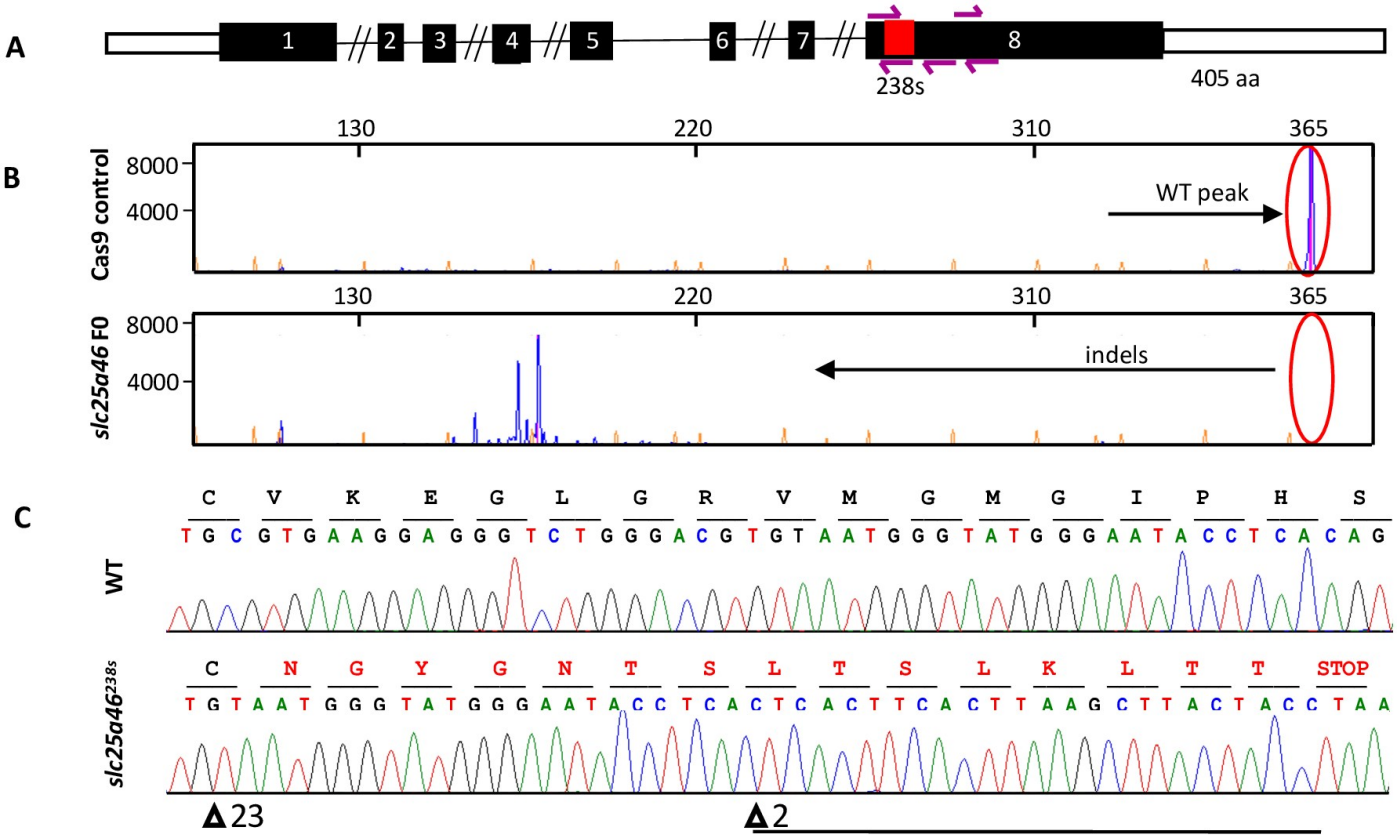
Genome editing of *slc25a46* gene in zebrafish using CRISPR/Cas9 system: (A) Schematic diagram of zebrafish slc25a46 gene with a frameshift in exon 8 indicated as a red box and a premature stop codon at amino acid position 238 (allele *slc25a46*^*238s*^). The magenta arrows indicate five CRISPR guides injected together to create both F0 crispants and stable mutants. (B) Fragment analysis of the amplified CRISPR targeted region from the FAM-labeled PCR product (365 base pair amplicon) showing WT peak in control and multiple peaks indicating insertions and deletions (indels) in a representative *slc25a46* F0 sample. X-axis represents base pair number; Y-axis represents signal intensity. (C) Sequence traces of wild-type (WT) and *slc25a46*^*238s*^ stable mutant zebrafish: exon 8, residues 223–238 indicating homozygous frameshift (in red); deletions are indicated with a triangle, insertion is underlined.

To generate a stable loss-of-function zebrafish model of *slc25a46* we first created F0 CRISPR mosaic mutants, or crispants. We designed five non-overlapping single guide RNAs (sgRNA) targeting the beginning of the largest exon 8, which encodes a conserved mitochondrial substrate carrier domain (IPR018108, InterPro)[44, 45] (Fig 1A). We also targeted this domain because most of the disease-causing mutations [30, 31, 44], and a frameshift in a mouse model, which developed ataxia, optic atrophy and peripheral neuropathy, reside in exon 8[33]. Finally, by choosing exon 8, we minimized the chances of functional restoration of the protein by alternative mRNA processing[46]. The pool of sgRNAs complexed with the Cas9 protein and injected into the zebrafish embryos at one-cell stage induced efficient mosaic mutagenesis (Fig 1B; lower trace). The pattern consisted of insertions, substitutions and deletions (indels), which appear as multiple peaks of shorter product length on the traces of fragment analysis, with similar peaks in different individually mutagenized larvae. We estimated the F0 mutagenesis as highly efficient based on the comparison between the fluorescent signal intensity of the peaks indicating the wild-type (WT) fluorescent PCR product of the CRISPR targeted region (365 base pairs) and multiple shorter product peaks representing a combination of stochastic indels[47]. We observed a common disappearance of the WT peaks amplified from individually genotyped F0 larvae (on average, in 80% of injected embryos). While it was not possible to immediately evaluate whether observed indels were in frame or out of frame using fragment analysis, we hypothesized that such a disruption of a conserved sequence site would likely have deleterious impact to the protein function. Although most F0 larvae did not survive to adulthood, they were all given an equal opportunity to grow, which minimized any founder effects but did not exclude the possibility of natural selection for genetic modifiers. The remaining F0 mosaic adult founders were used to cross with WT fish and propagate F1 heterozygous generation with one type of mutated allele per fish which were again raised in batches in which competition and natural selection for genetic modifiers could come into play. When we Sanger sequenced heterozygous F1 *slc25a46* progeny, we were able to identify the most common types of mutations induced in *slc25a46*. Among those were multiple indels that altered a stretch of 17 amino acids (aa) before inducing a premature stop codon which would be predicted to produce a protein half the WT length: 238 out of 405 aa (Fig 1C). We selected the heterozygous F1 founders with a mentioned frameshift for an in-cross and grew F2 homozygous progeny, followed by an F3 and F4 generations. We refer to this allele as *slc25a46*^*238s*^, and experiments reported hereafter were performed on the F4 larvae, referred to as mutants.

Due to the absence of a reliable antibody, it was not possible to validate the protein levels in mutant zebrafish by western blot. We did Sanger-sequence cDNA made from mutant mRNA and we confirmed that it is the same as the genomic DNA sequence, eliminating the possibility of transcriptional alterations (S1 Fig). Additionally, although the quantitative real-time polymerase chain reaction (RT-PCR) did not show a notable decrease in either *slc25a46* crispant or mutant transcript abundance (S2 Fig), it is likely that the truncated protein was quickly degraded due to its conformational instability, previously shown to adversely affect the protein in the targeted region[30, 31, 33, 35, 39, 44].

### CRISPR/Cas9 mutagenesis of *slc25a46* causes motoneuron defects in F0 crispant but not in mutant larvae

**Fig 2:**
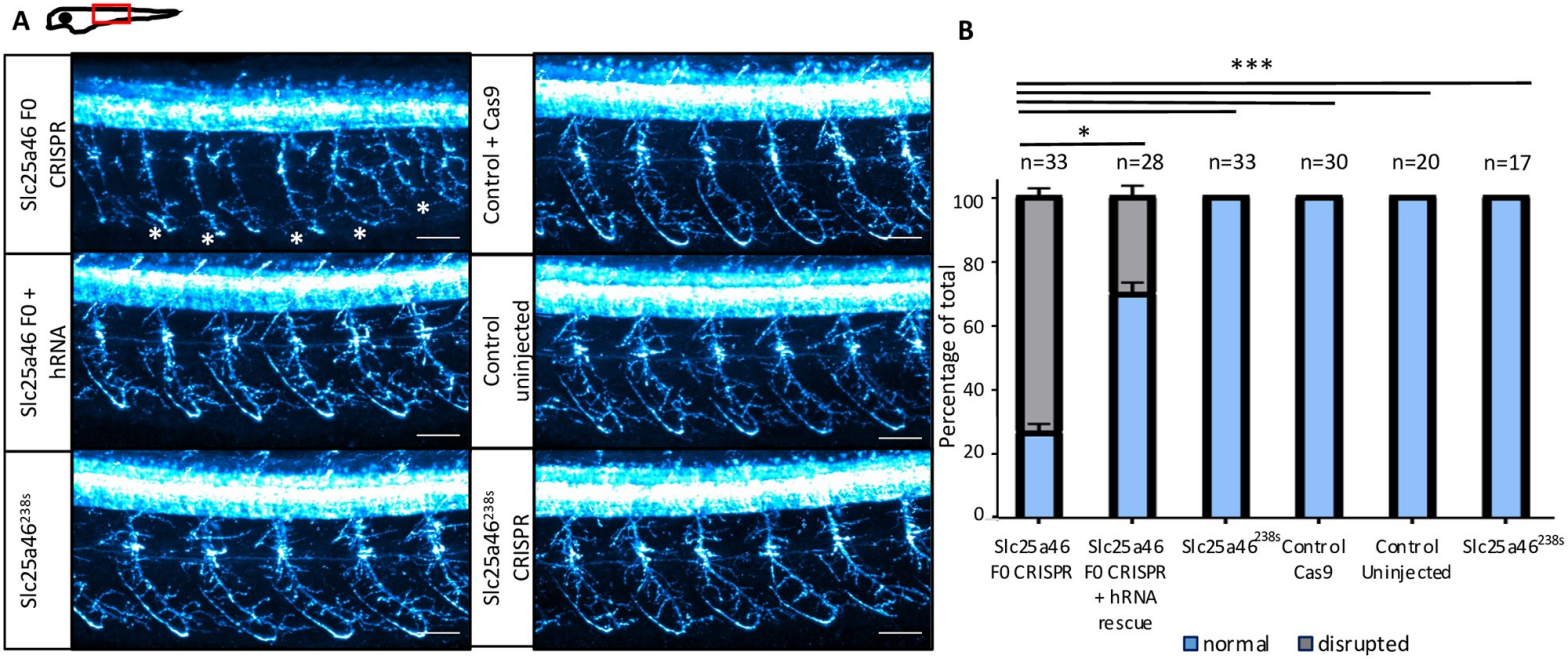
CRISPR/Cas9 targeting of *slc25a46* gene in zebrafish leads to significant motoneuron axon disruption in *slc25a46* F0 but not in *slc25a46*^*238s*^ zebrafish: (A) Confocal micrographs of 48 hpf zebrafish motoneurons stained with znp1 (cyan), whole mount, Z stack, lateral view captured above the yolk extension. Scale bar = 50 um. (B) Qualitative assessment of the primary motoneuron axon phenotypes: normal represent stereotypical “hook-like” axon path as in control images; disrupted–any number of abnormal motoneuron axons, such as axonal projections crossing into a nearby segment, truncated axons with projections not reaching back up to form the hook shape or aberrant hooks missing stereotypical branching pattern (indicated by white asterisks). P-values for comparisons between phenotypic penetrance in different genotypes are calculated by Fisher’s exact test (* for p<0.05; *** for p<0.001); n represents the number of individual larvae with observed motoneuron phenotype. Error bars represent SEM.

**Fig 3:**
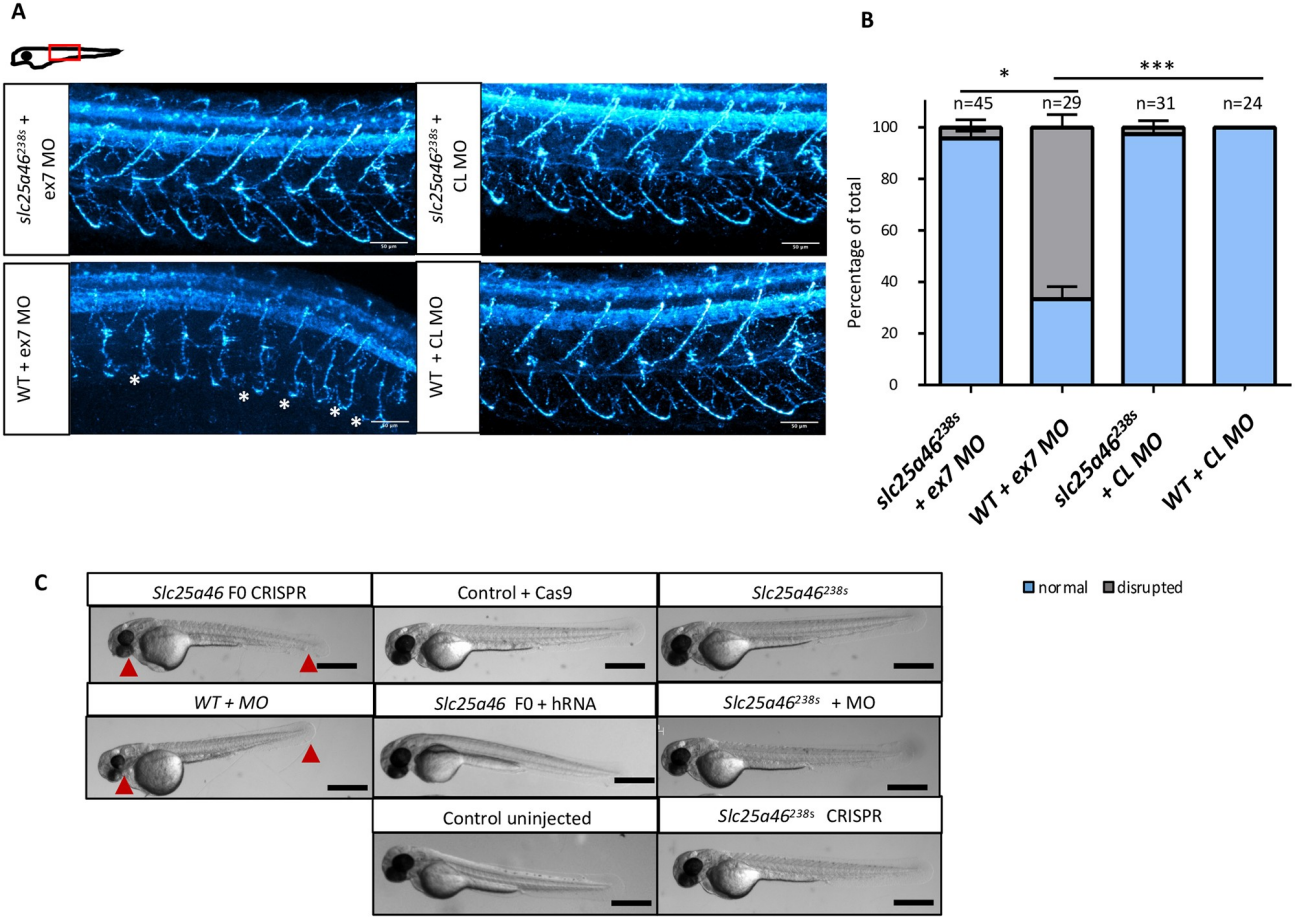
*slc25a46*^*238s*^ zebrafish are more resilient to *slc25a46* morpholino (MO) injections than WT controls: (A) Confocal micrographs of 48 hpf zebrafish motoneurons stained with znp1 (cyan), whole mount, Z stack, lateral view captured above the yolk extension. Scale bar = 50 um. (B) Qualitative assessment of the motoneuron axon phenotypes: normal represent stereotypical “hook-like” axon path as in control images; disrupted–any number of abnormal motoneuron axons, such as axonal projections crossing into a nearby segment, truncated axons with projections not reaching back up to form the hook shape or aberrant hooks missing stereotypical branching pattern (indicated by white asterisks). P-values for comparisons of phenotypes between genotypes is calculated by Fisher’s exact test (* for p<0.05; *** for p<0.001). N represents the number of individual larvae with observed motoneuron phenotype. Error bars represent SEM. (C) Brightfield micrographs of 48 hpf zebrafish larvae, lateral view. Images represent the most commonly observed phenotype within a group. Scale bar = 0.5 mm. Red arrows indicate smaller eyes, eye coloboma, shorter trunk in phenotypically distinct zebrafish.

To determine whether CRISPR induced genetic lesion in F0 larvae recapitulated the previously reported morpholino knockdown phenotype we evaluated the primary motoneuron axons at 48 hpf for any abnormalities[31]. To visualize the motoneurons, we performed whole mount immunostaining with znp1 antibody and utilized confocal microscopy focused over the lateral region of the spinal cord above the yolk extension[48]. Indeed, we observed a range of phenotypes similar to those induced by a morpholino, which we subdivided into the following categories: normal represent expected “hook-like” axon path as in control images; disrupted–two or more axons have an abnormal path. (Fig 2A). Using Fisher’s exact test, we showed that *slc25a46* F0 crispants had significantly higher occurrence of motoneuron axon disruptions (p = 0.001) than Cas9 injected controls (Fig 2B and S5 Table). To test for the specificity of the genetic disruption we co-injected an in-vitro synthesized human *SLC25A46* mRNA (hRNA) with intention to rescue the observed motoneuron phenotype. The significant reduction in axonal abnormalities (p = 0.002) confirmed the specificity of the *slc25a46* F0 CRISPR motoneuron phenotype (Fig 2A & 2B). In addition, the F0 larvae often had gross phenotypes similar to morphants, specifically smaller eyes, heart edema and shorter trunk size, which were also rescued by *SLC25A46* hRNA (Fig 3C). Furthermore, the F0 motoneuron and gross body abnormalities rescuable by hRNA supplementation serve as experimental evidence of severity of exon 8 disruption and significant protein destabilization. The gross F0 phenotypes (edema, smaller eyes, shorter trunk) observed at 2 dpf were mostly recovered by the feeding stage of 6–7 dpf, and larvae were able to swim and balance while swimming normally.

In contrast to *slc25a46* crispants, the *slc25a46*^*238s*^ mutants showed neither drastic motoneuron disruption (Fig 2A & 2B), nor heart edema, smaller eyes or shorter trunk (Fig 3C). The motoneuron length was also not significantly altered in *slc25a46*^*238s*^ mutants as evaluated by a neurite tracer method (S3 Fig). Examination of F2 mutants suggests compensation started in this generation as F2 slc25a46-/- larvae were indistinguishable from their WT or heterozygous siblings, not showing clear phenotypes such as those observed in F0 crispants.

To further rule out the possibility of off-target effects potentially causing the observed F0 CRISPR phenotype we injected mutant embryos with the same mix of pooled sgRNAs and Cas9 protein, while knowing that due to the change in genomic sequence *slc25a46*^*238s*^ embryos were lacking the original PAM sites necessary for CRISPR gene editing. We observed no motoneuron defects in CRISPR injected *slc25a46*^*238s*^ larvae at 48 hpf, which additionally confirmed that motoneuron defects observed in F0 CRISPR were specific and not due to nucleotide toxicity or other off-target effects (Fig 2A & 2B).

### *Slc25a46*^*238s*^ mutants are resilient to *slc25a46* morpholino knockdown

We next set out to test the hypothesis that *slc25a46*^*238s*^ mutants adapted to a deleterious mutation via genetic buffering by challenging both WT and mutants with *slc25a46* morpholino, following previously employed methodology for mutants displaying genetic compensation[10, 12]. We first injected WT and mutant embryos with a previously validated morpholino against the splice site of exon 3 (ex3 MO) [31] (S1 Table). We aimed to replicate a published ex3 MO phenotype by injecting a reported dose and compared it to un-injected controls. We evaluated the larvae at 48 hpf and observed significantly larger number of embryos with disrupted motoneurons (S5 Fig) in the WT group injected with morpholino versus the mutant larvae. A significantly smaller percent of mutant embryos showed a similar motoneuron phenotype (S5A & S5B Fig). Hypothesizing that such effects were produced by non-specific nucleotide toxicity[22], we then tested a newly designed morpholino against the splice site of exon 7 (ex7 MO) alongside with standard control morpholino (CL MO). To confirm the transcriptional consequences of ex7 MO, which is designed to cause a subsequent frameshift, we performed RT-PCR and observed a shorter band of predicted size in ex7 MO injected embryos compared to CL MO (S6 Fig), which infers exon 7 skipping. We evaluated the motoneuron phenotype by immunostaining and observed a large percent of disrupted motoneurons in WT embryos injected with ex7 MO, but not in *slc25a46*^*238s*^ mutants (Fig 3A & 3B). The disappearance of disrupted motoneuron phenotypes in the mutant group suggested reduced nucleotide toxicity associated with the ex7 MO (Fig 3A & 3B). Statistical significance was evaluated by Fisher’s exact test (Fig 3B and S5 Table). The decreased sensitivity of the *slc25a46*^*238s*^ mutant to morpholino knockdown compared to control demonstrates that the lack of phenotype in the mutant is not likely to be due to remaining *slc25a46* function.

#### *Slc25a46*^*238s*^ mutants show distinct gene expression profiles compared to F0 or control

**Fig 4:**
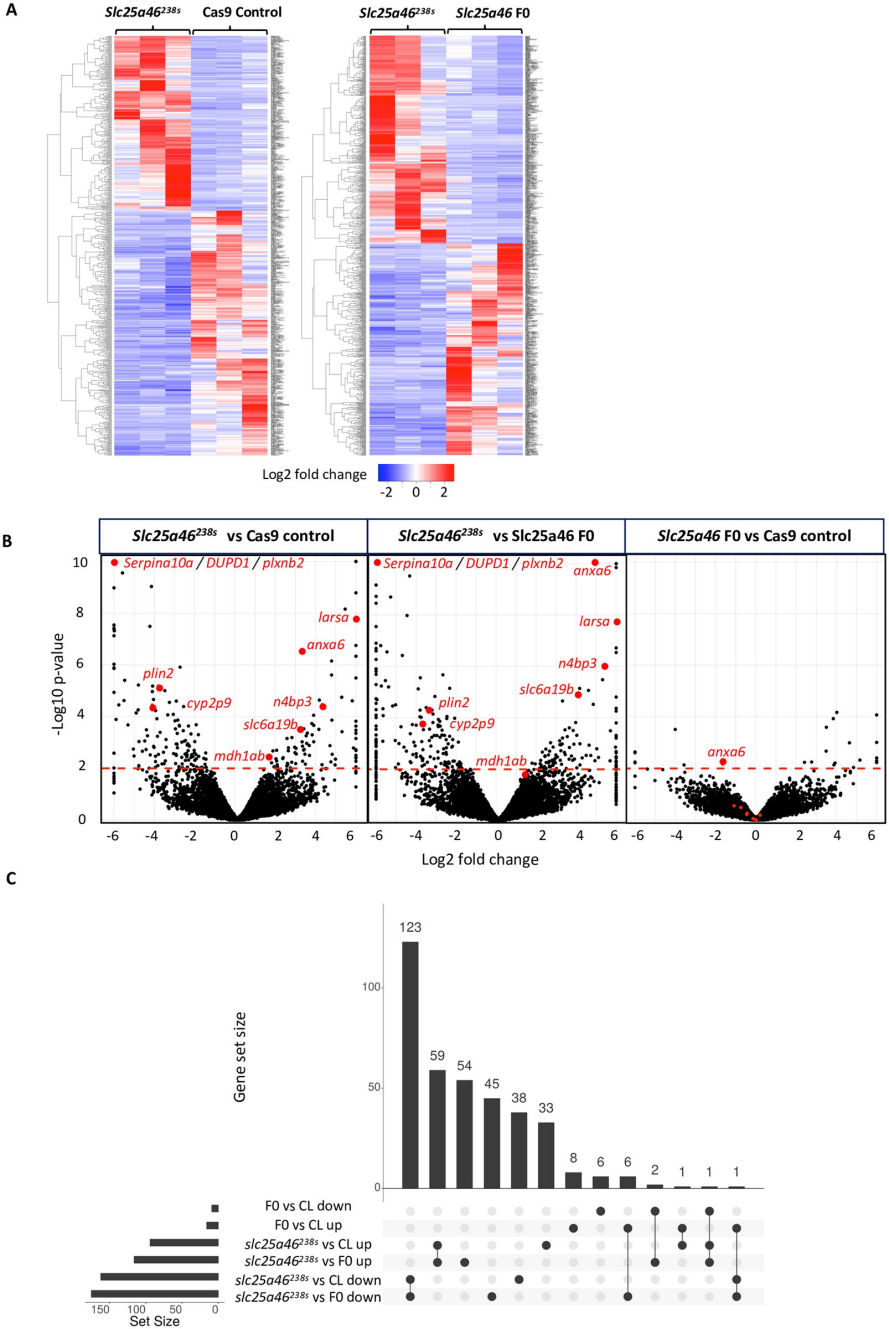
*Slc25a46*^*238s*^ zebrafish show a number of differentially expressed genes based on mRNA sequencing data: (A) Heat maps of all genes mapped to zebrafish genome comparing *slc25a46*^*238s*^ mutant to Cas9 control and *slc25a46* F0 CRISPR; (B) Volcano plots showing differentially expressed genes between *slc25a46*^*238s*^ mutant versus Cas9 control or *slc25a46 F0* compared to Slc25a46 F0 versus Cas9 control. Points above the red dashed line represent differentially expressed genes with p≤0.01; Red points represent chosen top genes of interest; (C) Upset plot showing intersections of upregulated and downregulated genes common (connected dots) and uniquely differentially expressed (individual dots) in *slc25a46*^*238s*^ mutant vs *slc25a46* F0 and Cas9 control. The plot is based on the genes with p≤0.01.

To test whether the lack of a phenotype in *slc25a46*^*238s*^ mutants could be due to compensatory changes in gene expression, we performed mRNA sequencing of whole embryos at 48 hpf and compared *slc25a46*^*238s*^ mutant’s gene expression to F0 crispants and unmutated controls injected with Cas9 protein only. We found a number of significantly differentially expressed genes in *slc25a46*^*238s*^ mutants compared to Cas9 controls (Fig 4A–4C), with 92 genes being upregulated and 161 being downregulated (p≤0.01). F0 crispants had many fewer changes compared to Cas9 control. Additionally, most genes found up-regulated (59) and down-regulated (123) in stable mutants compared to Cas9 control were also found similarly differentially regulated with the same directionality when compared to F0 crispants (p≤0.01) (Fig 4C). We speculate that the reason why we see more changes in mRNA expression in mutants rather than in crispants relates genetic compensation that is present in the mutants but not the F0, consistent with multiple previous reports[11, 19].

Approximately two thirds of the differentially regulated genes lacked complete annotation in the zebrafish genome, having sanger institute genomic sequence annotation with a prefix “si:” instead of a traditional gene name, and many highly dysregulated genes lack human (mammalian) orthologs in the Ensembl database (www.ensembl.org). Surprisingly, among annotated genes we did not see an upregulation in expression of any direct interactors of Slc25a46, such as Opa1 and Mfn2[38], or genes with high sequence homology and homologous domains, such as the other SLC25 family members, which could be expected to perform compensatory functions. We noted the up-regulated known genes with the lowest p-values and highest fold changes to be: Annexin A6 (*anxa6*), Leucyl-TRNA synthetase (*larsa*), NEDD4 Binding Protein 3 (*n4bp3*), Solute Carrier Family 6 Member 19 (*slc6a19b*), Membrane Spanning 4-Domains A8 (*ms4a17a*.*8*); and the down-regulated genes: Dual Specificity Phosphatase And Pro Isomerase Domain Containing 1 (*dupd1*), Serpin Family A Member 10 (*serpina10a*), Plexin B2 (*plxnb2*), Parathyroid Hormone 2 Receptor (*pth2r*), Perilipin 2 (*plin2*), Cytochrome P450 Family 2 Subfamily J Member 2 (*cyp2p9*) (Fig 4B). In particular, we found that *anxa6*, which belongs to annexin calcium binding protein family, was the only significantly down-regulated gene in F0 relative to Cas9 control, which was also significantly up-regulated in the stable mutant relative to Cas9 control (p≤0.01). Interestingly, *anxa6* was previously shown to be down-regulated in *Slc25a46* mutant mouse model, which maintained the disease phenotype across generations [35].

We then analyzed differentially expressed genes with Panther software (patherdb.org) and found protein classes which were differentially expressed in the stable mutant[49]. Among those classes was a high number of serine proteases and their transcription factors, serine protease inhibitors, DNA-binding proteins and a smaller number of transporters, immunity, and other proteins (S4 Fig). Overall, these data underscore a number of genetic changes, any of which could contribute to genetic buffering.

## Discussion

Genetic approaches that aim to functionally confirm putative disease genes frequently confront the absence of expected phenotypes in model systems. In addition, mismatches are common between knockdown and stable mutant phenotypes[50], raising concerns about the reliability of such functional models. The lack of a phenotype in stable mutant models can at least partially be explained by genetic compensation, though a thorough understanding of underlying mechanisms remains incomplete [19]. Investigation of such disease-phenotype-buffering pathways has the potential to lead to identification of therapies derived from compensatory mechanisms, while disease modeling approaches that minimize confounding effects of compensation will save the researchers both time and finances in addressing disease phenotypes.

In the current study, we report a case of efficient disease modeling in the first generation of zebrafish using CRISPR/Cas9 strategy and explore the potential pathways of genetic compensation in a stable *slc25a46*^*238s*^ mutant zebrafish. We show that mutants lack the motoneuron phenotype seen in both F0 crispants and morphants, suggesting that mutants have utilized buffering pathways lacking in F0 crispants. Consistent with this idea, through mRNA sequencing we identified over one hundred significantly dysregulated genes in mutants but not in crispants, some of which likely perform compensatory functions. A previously reported mechanism for genetic buffering was shown to be in the form of transcriptional adaptation elicited by NMD[9]. We observed no NMD for either crispant or *slc25a46*^*238s*^ mutants. Moreover, none of the dysregulated genes in mutants had sequence homology with the loss-of-function *slc25a46* supporting that the mechanism underlying compensation in *slc25a46*^*238s*^ is not triggered by NMD, but may involve the action of genetic modifiers or an orchestral work of multiple genes within relevant networks.

To explore which genes are most likely to buffer *slc25a46*^*238s*^ mutant phenotypes, we considered the normal functions of Slc25a46. Slc25a46 has been shown to be an important player in mitochondrial dynamics[31, 33]. As such, we expected to see changes in expression of genes involved in mitochondrial fission/fusion function, such as fission gene *drp1*, and fusion genes *opa1* or *mfn2*, or genes in the MICOS complex, previously reported to be dysregulated through proteomic analysis of an *Slc25a46* knockout mouse model[34, 35]. By contrast to the mouse model, we did not observe any significant changes in expression of transcripts of these genes.

Interestingly, we found *annexin a6* (*anxa6*) transcripts to be both significantly up-regulated in the *slc25a46* mutants, and significantly down-regulated in F0 crispants. This finding in zebrafish is consistent with the proteomics profile of *Slc25a46* knockout mice [35], which had the disease phenotype and in which Annexin A6 was among top downregulated proteins. Annexin A6 has a related but opposite effect to Slc25a46 on mitochondrial network state[51]. Annexin A6 was previously shown to be associated with the mitochondrial master regulator of fission Drp1, and can either inhibit or facilitate Drp1 mitochondrial localization in the context of elevated intracellular calcium[51]. In zebrafish, *anxa6* is largely enriched in the muscle tissue, while *slc25a46* is enriched in the CNS and PNS, however, both genes are expressed in all these tissues (www.proteinatlas.org)[52]. Although an interesting speculation, *anxa6* remains only one candidate for genetic compensation, possibly acting as a genetic modifier. Further investigation will be necessary to elucidate the relationship between *anxa6* and *slc25a46*, as well as their interplay in the mitochondrial morphogenesis.

Many other genes in addition to *anxa6* show dysregulation in the stable *slc25a46* mutants. These findings highlight that our understanding of the normal function of Slc25a46 is incomplete. Apart from being involved in mitochondrial architecture maintenance, Slc25a46 has also been implicated in ER tethering and phospholipid transfer between ER and mitochondria[38]. It will be crucial to conduct further functional studies in order to unravel the potential compensatory effects of several other dysregulated genes on the developmental morphogenesis of motoneurons, such as Leucyl-TRNA synthetase (*larsa*), which loss is observed in hepatic pathologies and metabolic malfunction[53]; NEDD4 Binding Protein 3 (*n4bp3*) involved in axonal guidance and dendritic arborization[54]; Plexin B2 (*plxnb2*): semaphorin receptor implicated in axonogenesis and angiogenesis[55]. Taken together, while they do not resemble previously reported NMD induced gene expression changes, the mechanisms underlying genetic compensation reported in our study are yet to be elucidated.

In summary, our study presents a case of efficient disease modeling in the first generation of zebrafish using CRISPR/Cas9 strategy and explores the potential pathways of genetic compensation in a stable *slc25a46*^*238s*^ mutant zebrafish, which mechanisms appear to involve an overall response of the genes within metabolic and developmental gene networks, likely being a result of sensing and adapting to the altered metabolic needs. Importantly, we provide the genetics field with a case of effective F0 CRISPR mutagenesis, which in combination with carefully designed control experiments supports the feasibility of the F0 CRISPR approach to modeling genetic loss-of-function candidate disease genes.

## Methods

### Zebrafish husbandry

Experiments were carried out using transgenic line *Tg(aldoca*:*gap43-Venus*)[56]. Adults were kept on a 14-h light/10-h dark cycle at 28 °C. Embryos were collected from natural crosses after removing a divider at the beginning of the light cycle. Embryos were raised in Petri dishes in system water at 28 °C under standard conditions. For live imaging (bright field) and sacrifice for whole mount immunostaining at 48 hpf embryos were anesthetized with 0.02% tricaine methanesulfonate (Sigma). All experiments were conducted in accordance with University of Miami Institutional Animal Care and Use Committee guidelines and are described in approved protocol #18–128.

### sgRNA design and synthesis

sgRNAs were chosen from among top targets identified by CHOPCHOP software (http://chopchop.cbu.uib.no/) with NGG PAM sites and zero predicted off-targets (with fewer than three mismatches in the *slc25a46*-targeting 20-mer). All sgRNAs were designed against the beginning of exon 8 (S1 Table). sgRNAs were generated by the oligonucleotide assembly method as described in[57]. RNAs were synthesized using the HiScribe^™^ T7 Quick High Yield RNA Synthesis Kit (New England Biolabs) with an incubation time of 12 h for the *in vitro* transcription reaction. RNAs were purified with the RNA Clean & Concentrator^™^-5 kit (Zymo Research), eluted with 15 μl water and diluted to working concentrations ~ 400 ng/μl.

### Microinjections

Microinjections were performed at one-cell stage and Cas9 and nucleotides were diluted in nuclease free water with 1% Fast-Green (Sigma). Cas9 protein (PNA Bio) reconstituted in nuclease-free water was mixed with five pooled sgRNAs. The mixture was incubated for 5 min at 37 degrees Celsius. For the rescue injections mixtures were supplemented with synthesized wild-type human *SLC25A46* RNA (hRNA). Cas9 protein diluted in nuclease-free water and 1% Fast-Green dye was used for control injections. Injection needles were calibrated to dispense 0.5 nL of the mixture. Approximately 1.5 nL of active sgRNA-Cas9 ribonucleoprotein complex plus hRNA were injected per embryo into the cell. The final amounts injected per embryo approximately were: 315 picograms (pg) of Cas9 protein; 350 pg of sgRNA pool; 40 pg of hRNA. At least three independent injection experiments were performed with spawns from different founder fish to control for batch effect.

Morpholino injections were done according to suggested guidelines for the use of morpholinos[58]. Approximately 1.5 ng of both exon 7 *slc25a46* and standard control morpholino (diluted 1:2) obrained from Gene Tools was injected into the cell of the embryos at one-two cell stages (See S1 Table for the morpholino sequence and RT-PCR primers). RT-PCR (Superscript IV One step RT-PCR system, ThermoFisher Scientific) was performed using exon 5 forward primer and exon 8 reverse primer (S1 Table) on RNA extracted from 48 hpf pooled embryos. Exon 3 morpholino was used undiluted; approximately 0.5 ng was injected into the cell at one cell stage.

### Whole-mount immunofluorescence

Zebrafish larvae were dechorionated at 48 hpf (hours post fertilization) and fixed in 4% paraformaldehyde and phosphate-buffered saline with 0.025% Triton X (PBTx) solution at room temperature for 4.5 hours. All washes and staining solutions were made and performed according to standard protocols. Tissues were permeabilized with cold (-20°C) acetone for 9 minutes. *Znp1* primary antibody raised in mouse (Zebrafish International Resource Center) was used for staining motoneurons, diluted 1:500, rocked at 4°C overnight. After 3 PBTx washes, secondary Donkey-anti-Mouse (633) antibody was used at a concentration 1:500, rocked at 4°C overnight.

### Confocal imaging of motor neurons

Motoneuron outgrowth was assayed at 48 hpf. Immunostained zebrafish were mounted laterally in 1% low-melting point agarose and imaged using a Leica confocal microscope with a 20× air lens. 1-μm thick *z* stacks were collected between myotome segments 6 and 13[59], and the motoneuron morphology was evaluated for its normal hook shape and outgrowth trajectory. Images were processed with Fiji software (ImageJ). Cyan filter was used to generate the figures.

### CRISPR efficiency testing by fragment analysis

Embryos were euthanized and DNA was extracted using 50 mM NaOH digestion at 95 degrees Celsius for 30 min. Diluted 1:5 DNA then was used to run Fluorescent PCR as described in [57]. The reaction products were run on a Genetic Analyser 3130xl using POP-7 polymer and analyzed for the disruption of the wild-type peaks as described in [47]. Fragment analysis was performed on single embryos.

### Statistical analysis of motoneuron phenotypes

Images from three separate experiments were blindly evaluated for qualitative inclusion into either “normal” group or “disrupted” group. The normal group was assigned to images with stereotypical motor axons shaped into normal hooks as on the images of uninjected controls[60]. The affected group was assigned to images with any number of disrupted motoneuron axons, such as axonal projections crossing into a nearby segment, truncated axons with projections not reaching back up to form the hook shape or aberrant hooks missing stereotypical branching pattern[60, 61]. The Exact Fisher’s test was performed for these two groups and the p value was calculated accordingly using GraphPad Prism software. For the statistical power calculation, we used G power software. All sample sizes, proportions, and effect sizes were included in S5 Table.

For the axon length measurements (S3 Fig) the Fiji (ImageJ) “Simple neurite tracer" was used[62]. The statistical significance was calculated by 2-tailed Student’s T test.

### RNA extraction

Total RNA was isolated from 48 hpf whole larvae from four separate batches of injected embryos. The samples used for mRNA isolation were first frozen at minus 80 degrees C, then extracted altogether with Direct-Zol RNA miniprep kit (Zymo). The concentration and purity of total RNA were determined using BR-RNA Qubit assay kit (Invitrogen). Amplification grade DNAse I (Invitrogen) was used for DNA traces degradation in RNA samples.

### cDNA Synthesis for genotyping of heterozygous and homozygous mutants

RT-PCR was performed using 0.1–5 μg of total RNA to synthesize the first strand of cDNA using First strand synthesis Reverse Transcriptase kit (Invitrogen). PCR amplification was carried out using the forward and reverse primers designed for exon 8 (S1 Table). The resulting PCR product was ligated into a plasmid vector TOPO TA cloning for sequencing (ThermoFisher Scientific). The plasmid construct was then transformed into One Shot^®^ TOP10 competent cells (Invitrogen), and the cells were plated on LB/ampicillin plates. The colonies were screened by colony PCR for the presence of the correct insert. Sanger sequencing of six different clones was carried out.

### Real-time quantitative PCR

cDNA was synthesized from approximately 1 μg of total RNA using First strand synthesis Reverse Transcriptase kit with random oligos (Invitrogen). Quantitative RT-PCR was performed using SYBR-Select Master mix (Applied Biosystems) with primers listed in the S1 Table, according to the protocol recommended by the manufacturer on a Quant Studio 12 K Flex machine. The PCR conditions consisted of an initial denaturation step at 95°C for 10 min followed by 40 cycles at 95°C for 15 s (denaturation) and 60°C for 1 min (elongation). The fold-changes were calculated relative to internal control *eef1a1* expression. A minimum of three biological and technical replicas were used to generate SEM.

### RNA sequencing

RNA sequencing library was prepared according to KAPA mRNA Hyper prep kit Illumina platforms protocol with the following parameters: initial RNA input was 100 ng; fragmentation step (3.3) was 6 min at 94°C; amplification step (9.3) was 14 cycles. The final libraries were pooled at a loading concentration of 2.0 nM and run on the NovaSeq 6000 using an SP flow cell and run in Standard Mode, SE100 (single end, 100 cycles). Four biological replicas were used in sequencing, out of which three replicas with the highest correlation scores were used for the subsequent analysis.

After quality control, raw reads were trimmed using trim galore to remove Illumina adapters and bases below a sequencing quality of 30. Reads were then aligned to *Danio rario* transcriptome (GRCz10, Ensembl.org) and quantified using STAR[63]. Differential expression was calculated using edgeR and significant genes were taken to be those below a p-value of 0.05[64].

Heatmaps were generated in R using heatmap2 and the Z-scores of expression per each gene. Red indicates higher expression and blue indicates lower expression.

Volcano plots were generated in R using ggplot2. The X-axis is the log base 2 of the fold change and the Y-axis is the -log base 10 of the P-value generated using edgeR[64]. Red dashed line indicates the level of significance by a p-value threshold of 0.01. Genes where 0, 1, or 2 samples of the higher expression group were below 0.1 Triplicates per million (TPM) were excluded from the graphs.

UpSet plots were generated in R using UpSetR. Only genes with a p-value less than or equal to 0.01 were used. Vertical bars show intersection sizes, while connected dots show which groups are being compared. Horizontal bars on the bottom-left show the complete size of each set of genes.

All RNA sequencing files are deposited to GEO database; GEO accession number GSE138414.

## Supporting information

S1 Fig Sanger sequence of *Slc25a46*^*238s*^ cDNA: Exon 8 beginning from the indel site induced by the first gRNA and ending with the indel induced by the last out of five gRNAs. The sites where gRNAs would bind in a WT sequence are indicated by the blue arrow: “+” strand on top of the sequence, “-”strand on the bottom. Deletions are indicated with a triangle and a base pair number, insertions are underscored.
(PDF)

S2 Fig Real time quantitative PCR in *slc25a46*^*238s*^ zebrafish: (A) *Slc25a46* mRNA levels in *slc25a46*^*238s*^ and F0 CRISPR mutant zebrafish at 48 hpf normalized to Cas9 control and compared to *slc25a46* morpholino (MO) knockdown; (B) *Anxa6* mRNA levels in *slc25a46*^*238s*^ and F0 CRISPR mutant zebrafish at 48 hpf normalized to Cas9 control; (C) *Serpina10a* and *mdh1ab* mRNA levels in *slc25a46*^*238s*^ and F0 CRISPR mutant zebrafish at 48 hpf normalized to Cas9 control; (D) *N4bp3 and slc6a19b* mRNA levels in *slc25a46*^*238s*^ and F0 CRISPR mutant zebrafish at 48 hpf normalized to *slc25a46*^*238s*^ mutant. Error bars in all graphs represent SEM.
(PDF)

S3 Fig Primary motoneuron axon length in *slc25a46*^*238s*^ mutants at 48 hpf: Motoneuron length was evaluated by Fiji ImajeJ plugin”simple neurite tracer”; n = 16 zebrafish in each group with 4 axons traced per fish from the same region above the yolk extension; p-value is calculated by 2-tailed Student’s T test; NS = non-significant. (PDF)

S4 Fig Protein classes of significantly dysregulated genes in *slc25a46*^*238s*^ mutants compared to Cas9 control based on mRNA sequencing data: Pie charts of protein classes identified by Panther database (www.pantherdb.org) among its hits for genes with p≤0.01 (45 hits out of 63 input IDs in the list of up-regulated genes; 66 hits out of 111 input IDs for in the list of down-regulated genes). (PDF)

S5 Fig *slc25a46*^*238s*^ zebrafish are more resilient to *slc25a46* morpholino injections than WT controls (additional exon 3 target): (A) Confocal micrographs of 48 hpf zebrafish motoneurons stained with znp1 (cyan), whole mount, Z stack, lateral view captured above the yolk extension. Scale bar = 50 um. (B) Qualitative assessment of the motoneuron axon phenotypes: normal represent stereotypical “hook-like” axon path as in control images; disrupted–any number of abnormal motoneuron axons, such as axonal projections crossing into a nearby segment, truncated axons with projections not reaching back up to form the hook shape or aberrant hooks missing stereotypical branching pattern (indicated by white asterisks). P-values for comparisons of phenotypes between genotypes is calculated by Fisher’s exact test. N represents the number of individual larvae with observed motoneuron phenotype. Error bars represent SEM.
(PDF)

S6 Fig RT-PCR confirms exon 7 skipping: Agarose gel showing bands of expected 520 bp size RT-PCR product in samples injected with CL MO, and a decreased band size in the samples injected with ex7 MO. (PDF)

S1 Table Primer, sgRNA and morpholino sequences. (PDF)

S2 Table *Slc25a46*^*238s*^ mutant vs *slc25a46* F0 CRISPR RNA sequencing results: TPM values, p-values, fold changes. (XLSX)

S3 Table *Slc25a46*^*238s*^ mutant vs Cas9 control RNA sequencing results: TPM values, p-values, fold changes. (XLSX)

S4 Table *Slc25a46*^*238s*^ F0 CRISPR mutant vs Cas9 control RNA sequencing results: TPM values, p-values, fold changes. (XLSX)

S5 Table Statistical data: Sample sizes and power analysis. Motoneuron phenotypes were quantified on batches of larvae from three different adult matings. These numbers are indicated below as total number of larvae with normal and disrupted phenotypes, with numbers in each batch indicated in parentheses.
(PDF)
