# Supplementary figures and images for: Genetic compensation in a stable slc25a46 mutant zebrafish: A case for using F0 CRISPR mutagenesis to study phenotypes caused by inherited disease
Source: PLoS One. 2020 Mar 24;15(3):e0230566. doi: 10.1371/journal.pone.0230566 (PMC7092968; doi:10.1371/journal.pone.0230566)

S1 Fig.

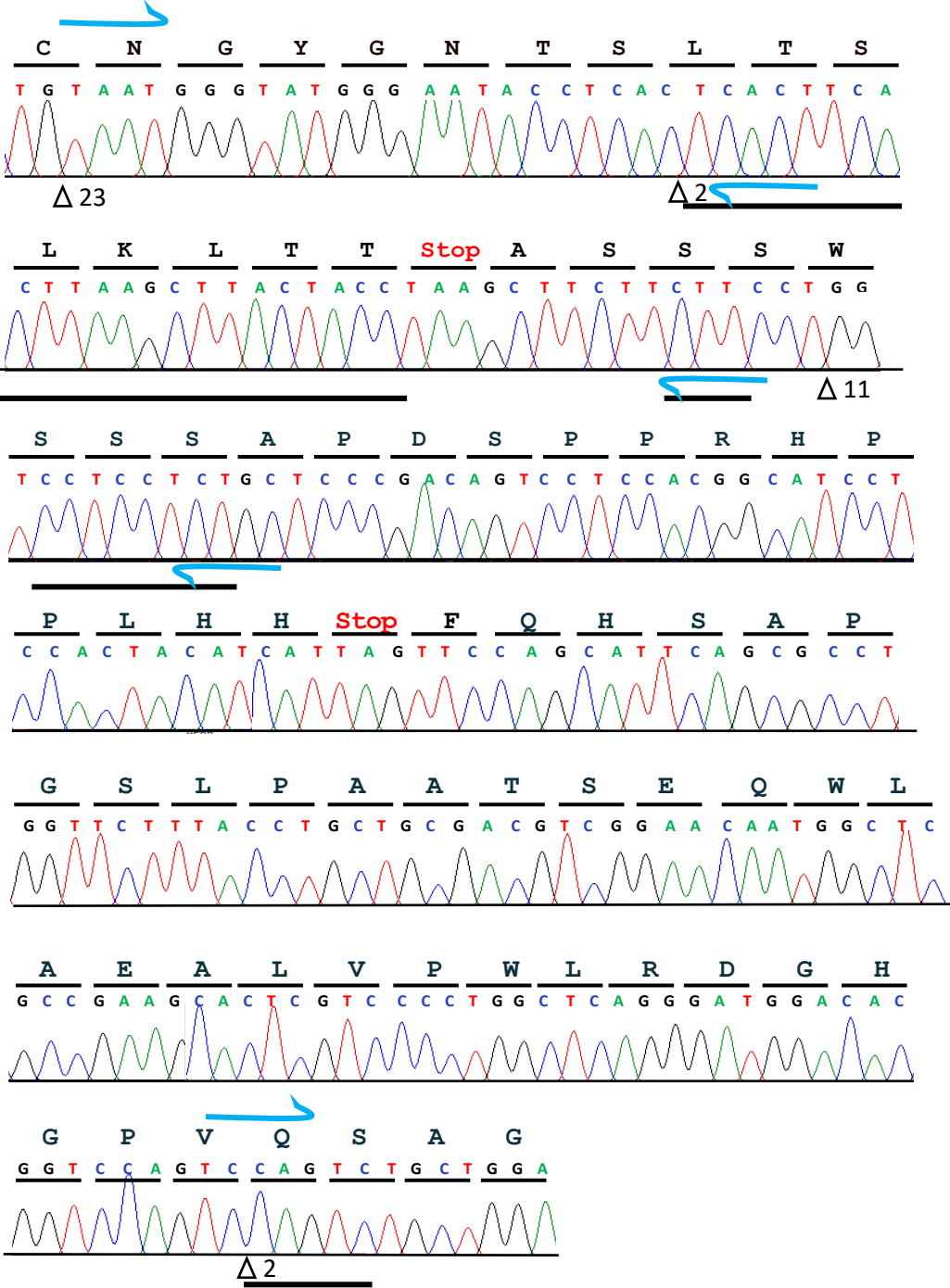

Supplement: S1 Fig — The sites where gRNAs would bind in a WT sequence are indicated by the blue arrow: “+” strand on top of the sequence, “-”strand on the bottom. Deletions are indicated with a triangle and a base pair number, insertions are underscored. (PDF) [file pone.0230566.s001.pdf]

S2 Fig.

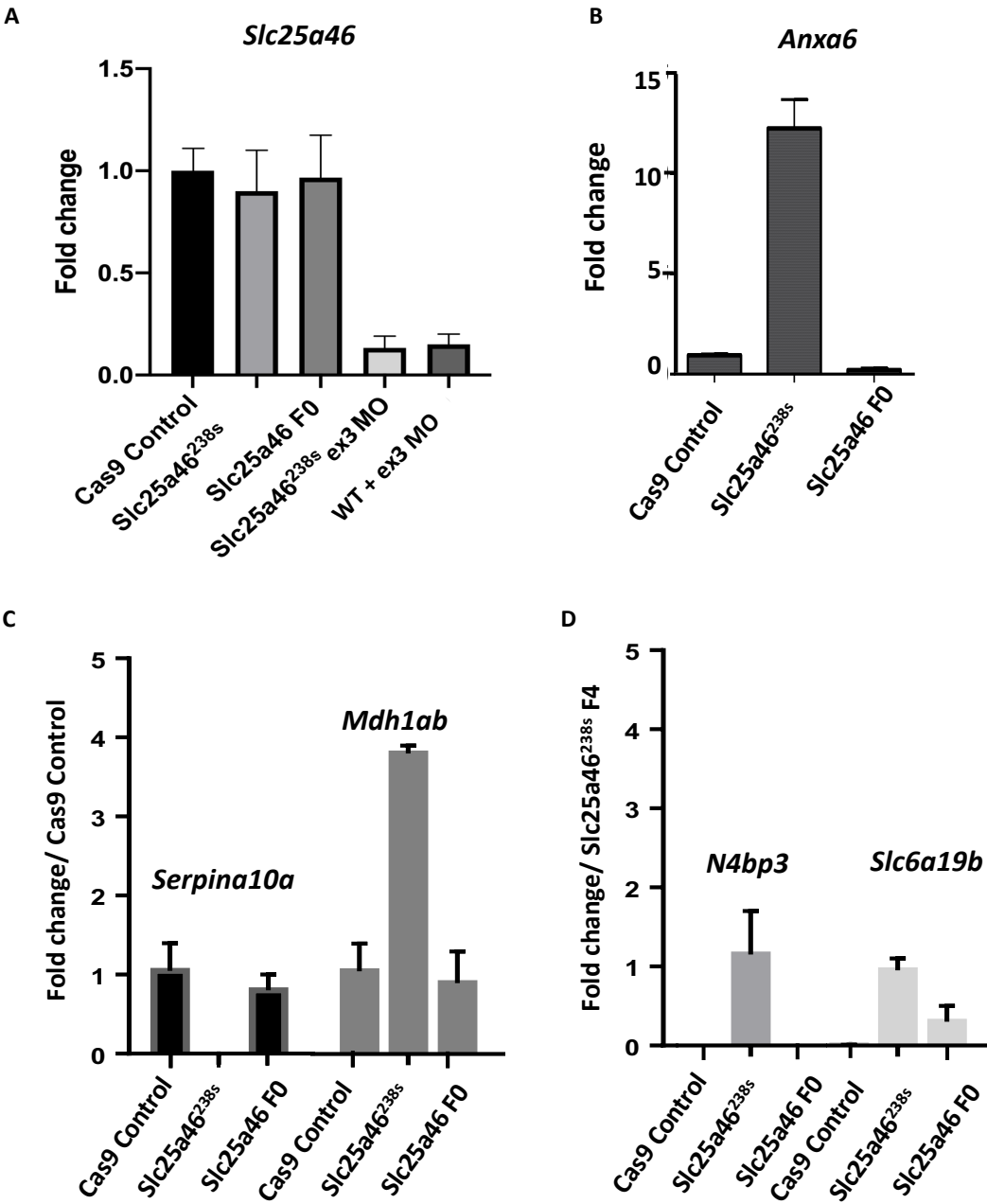

Supplement: S2 Fig — Real time quantitative PCR in slc25a46238s zebrafish: (A) Slc25a46 mRNA levels in slc25a46238s and F0 CRISPR mutant zebrafish at 48 hpf normalized to Cas9 control and compared to slc25a46 morpholino (MO) knockdown; (B) Anxa6 mRNA levels in slc25a46238s and F0 CRISPR mutant zebrafish at 48 hpf normalized to Cas9 control; (C) Serpina10a and mdh1ab mRNA levels in slc25a46238s and F0 CRISPR mutant zebrafish at 48 hpf normalized to Cas9 control; (D) N4bp3 and slc6a19b mRNA levels in slc25a46238s and F0 CRISPR mutant zebrafish at 48 hpf normalized to slc25a46238s mutant. Error bars in all graphs represent SEM. (PDF) [file pone.0230566.s002.pdf]

S3 Fig.

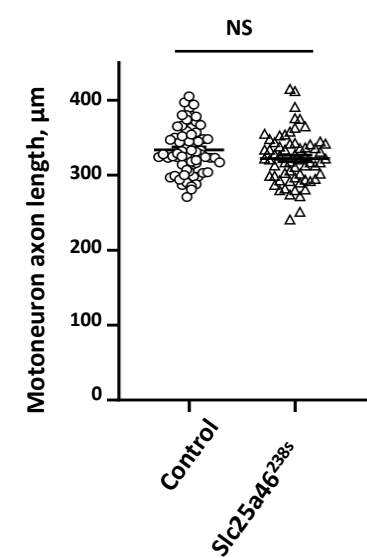

Supplement: S3 Fig — (PDF) [file pone.0230566.s003.pdf]

S4 Fig.

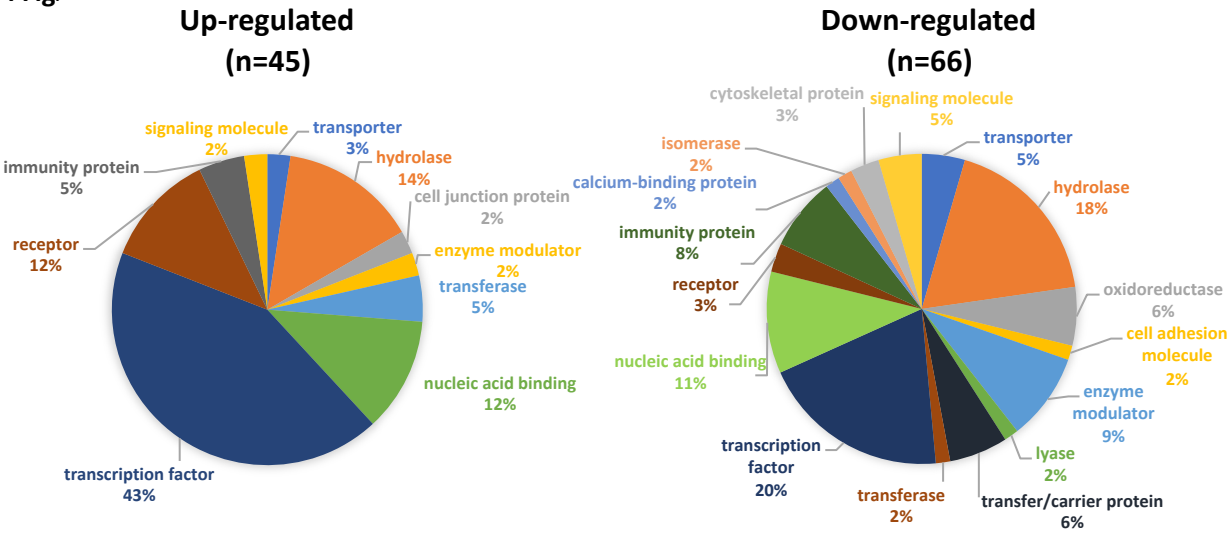

Supplement: S4 Fig — (PDF) [file pone.0230566.s004.pdf]

S5 Fig

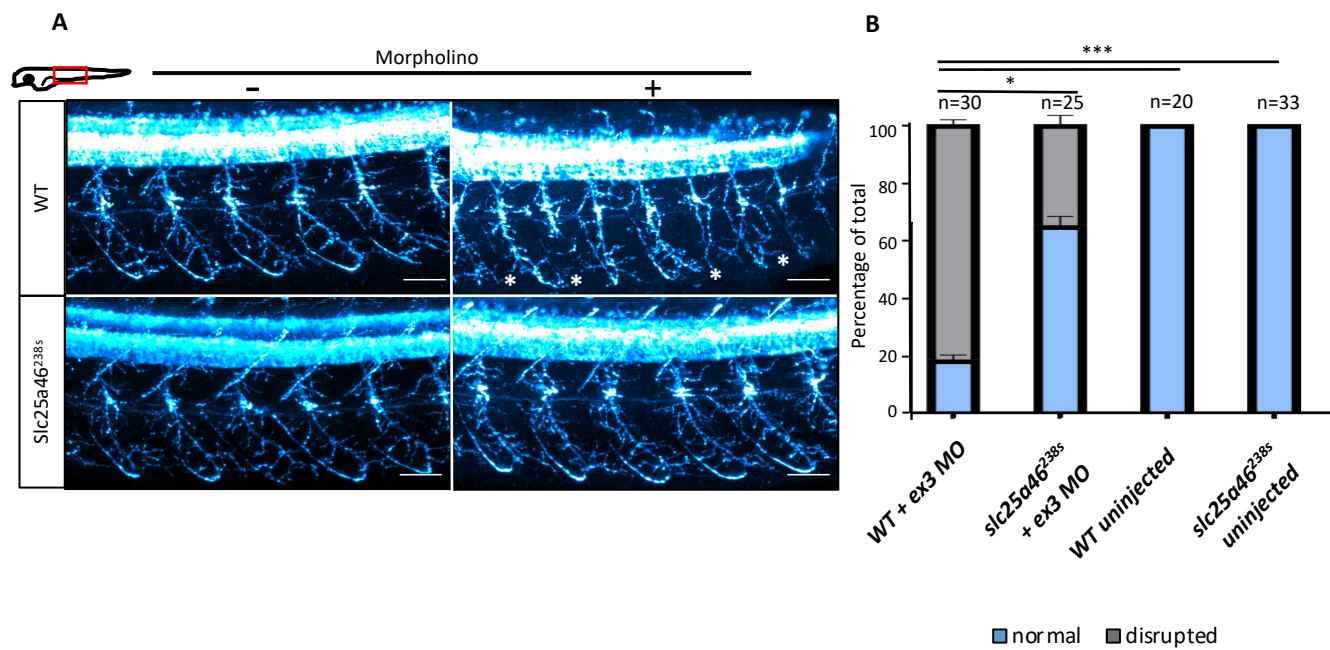

Supplement: S5 Fig — slc25a46238s zebrafish are more resilient to slc25a46 morpholino injections than WT controls (additional exon 3 target): (A) Confocal micrographs of 48 hpf zebrafish motoneurons stained with znp1 (cyan), whole mount, Z stack, lateral view captured above the yolk extension. Scale bar = 50 um. (B) Qualitative assessment of the motoneuron axon phenotypes: normal represent stereotypical “hook-like” axon path as in control images; disrupted–any number of abnormal motoneuron axons, such as axonal projections crossing into a nearby segment, truncated axons with projections not reaching back up to form the hook shape or aberrant hooks missing stereotypical branching pattern (indicated by white asterisks). P-values for comparisons of phenotypes between genotypes is calculated by Fisher’s exact test. N represents the number of individual larvae with observed motoneuron phenotype. Error bars represent SEM. (PDF) [file pone.0230566.s005.pdf]

S6 Fig.

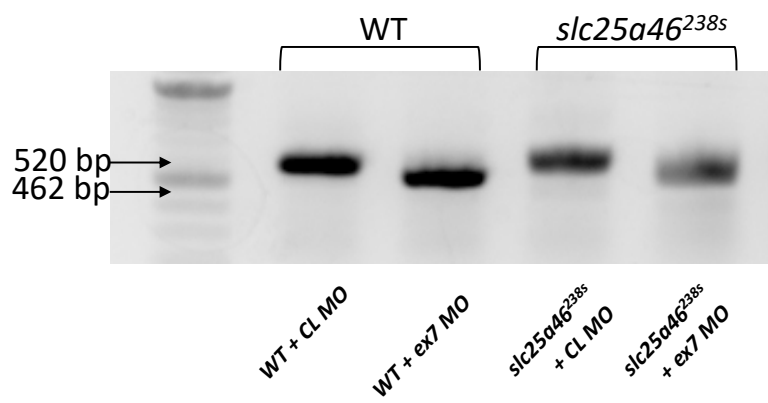

Supplement: S6 Fig — (PDF) [file pone.0230566.s006.pdf]
