# Supplementary material for: Genetic compensation in a stable slc25a46 mutant zebrafish: A case for using F0 CRISPR mutagenesis to study phenotypes caused by inherited disease
Source: PLoS One. 2020 Mar 24;15(3):e0230566. doi: 10.1371/journal.pone.0230566 (PMC7092968; doi:10.1371/journal.pone.0230566)
Supplement: S1 Table — (PDF) [file pone.0230566.s007.pdf]

S1 Table. Primers and Target sites.

**RT-PCR primers**		
**Gene_exon**	**Forward Sequence**	**Reverse Sequence**
n4bp3_exons 1-2	TGTCCCAATCTCTGGAGGAC	TTTCAGCTCCGTCACTTCCT
slc6a19b_exons 1-2	AAGCCCAATACATGCTGACC	AGTCCTCCAGACACCCACAC
mdh1ab_exons 1-2	CGTGAGGAAAATAGGGGTGA	CAATGCCGTACAGCAGAGAG
serpina10a_exons 2-3	AGAAGGTCAGGGATGTGGTG	AGAACGGAGCATCATGGGTA
Anxa6_exons 3-4	CGAGACCTGGAAGCAGATGT	GAGCATCTTCCTCCACAAGG
slc25a46_exons 3-4	CACCCCTGCATTGTGTTTC	GGTGACATTGTACATCACACTGA
**PCR primers for RT-PCR, Sanger and Fragment analysis**		
Slc25a46 - exon 8	tccagtcttcatccagtgtttc	CGGCAAAGCTGGCCATGAG
Slc25a46 - exon 5	GGAAAGGAATGGGAAGCACT	
**Slc25a46 Single Guides for CRISPR**		
**strand**	**Target sequence**	**PAM site**
-	GGGAGCACCAGGTTCCAGAG	AGG
+	GGACACGGTCCAGTCTATGC	TGG
-	GGAAGAAGACGCTTACTGTG	AGG
-	GGAGGACTGTCGGGAGCACC	AGG
+	GGAGGGTCTGGGACGTGTAA	TGG
**Morpholino**		
SLC25A46_MO_ex7_in7	AGTGTATCAAACCTCTTACCTGTAC	
