# Supplementary material for: Genetic compensation in a stable slc25a46 mutant zebrafish: A case for using F0 CRISPR mutagenesis to study phenotypes caused by inherited disease
Source: PLoS One. 2020 Mar 24;15(3):e0230566. doi: 10.1371/journal.pone.0230566 (PMC7092968; doi:10.1371/journal.pone.0230566)
Supplement: S5 Table — Motoneuron phenotypes were quantified on batches of larvae from three different adult matings. These numbers are indicated below as total number of larvae with normal and disrupted phenotypes, with numbers in each batch indicated in parentheses. (PDF) [file pone.0230566.s011.pdf]

S5 Table. Statistical measures and raw counts.

genotype / treatment		Motoneuron phenotype observed n (3 trials)		Total N	p (normal/total)
		normal	disrupted		
**Wild-type**	Slc25a46 F0 CRISPR	10 (3,2,3)	23 (7,8,10)	33	0.24 (0.3, 0.2, 0.23)
	Slc25a46 F0 CRISPR + hRNA	19 (6,6,7)	9 (4,3,2)	28	0.68 (0.6, 0.67, 0.78)
	Control Cas9	30 (10,10,10)	0 (0, 0, 0)	30	1 (1,1,1)
	Slc25a46 ex3 MO	5 (2, 2, 1)	25 (8, 8, 9)	30	0.17 (0.20, 0.20, 0.10)
	Slc25a46 ex7 MO	10 (5, 2, 3)	19 (7, 6, 6)	29	0.33 (0.42, 0.25, 0.33)
	Control MO	24 (9, 9, 6)	0 (0, 0, 0)	24	1 (1,1,1)
	uninjected	20 (6,6,8)	0 (0, 0, 0)	20	1 (1,1,1)
slc25a46^{238s}	Slc25a46 CRISPR	17 (6, 6, 5)	0 (0, 0, 0)	17	1 (1,1,1)
	Slc25a46 ex3 MO	16 (6, 6, 4)	9 (3, 2, 4)	25	0.64 (0.67, 0.75, 0.50)
	Slc25a46 ex7 MO	43 (18, 16, 9)	2 (1, 0, 1)	45	0.96 (0.97, 1, 0.9)
	Control MO	30 (8, 13, 9)	1 (0, 1, 0)	31	0.97 (1, 0.92, 1)
	uninjected	33 (10, 13, 10)	0 (0, 0, 0)	33	1 (1,1,1)

Comparison Configuration	p1 / p2	n1 / n2	actual α	Odds ratio	Power
slc25a46 F0 CRISPR	0.24	33			
slc25a46 F0 CRISPR + hRNA	0.68	28	0.03	0.15	0.95
slc25a46 F0 CRISPR	0.24	33			
WT Control Cas9	1	30	0	0	1
slc25a46 F0 CRISPR	0.24	33			
slc25a46^{238s} uninjected	1	20	0	0	1
slc25a46 F0 CRISPR	0.24	33			
Slc25a46^{238s} CRISPR	1	17	0	0	1
Slc25a46^{238s} ex3 MO	0.64	25			
WT ex3 MO	0.17	30	0.03	8.68	0.97
WT ex7 MO	0.33	29			
WT Control MO	1	24	0	0	0.99
Slc25a46^{238s} ex7 MO	0.96	45			
WT ex7 MO	0.33	29	0.05	44.57	0.99
